# Mitochondrial and Metabolic Dysfunction in Renal Convoluted Tubules of Obese Mice: Protective Role of Melatonin

**DOI:** 10.1371/journal.pone.0111141

**Published:** 2014-10-27

**Authors:** Alessandra Stacchiotti, Gaia Favero, Lorena Giugno, Antonio Lavazza, Russel J. Reiter, Luigi Fabrizio Rodella, Rita Rezzani

**Affiliations:** 1 Anatomy and Physiopathology Division, Department of Clinical and Experimental Sciences, University of Brescia, Brescia, Italy; 2 Istituto Zooprofilattico Sperimentale della Lombardia ed Emilia Romagna, OIE Reference Laboratory for RHD, Brescia, Italy; 3 Department of Cellular and Structural Biology, UT Health Science Center San Antonio, San Antonio, Texas, United States of America; The University of Manchester, United Kingdom

## Abstract

Obesity is a common and complex health problem, which impacts crucial organs; it is also considered an independent risk factor for chronic kidney disease. Few studies have analyzed the consequence of obesity in the renal proximal convoluted tubules, which are the major tubules involved in reabsorptive processes. For optimal performance of the kidney, energy is primarily provided by mitochondria. Melatonin, an indoleamine and antioxidant, has been identified in mitochondria, and there is considerable evidence regarding its essential role in the prevention of oxidative mitochondrial damage. In this study we evaluated the mechanism(s) of mitochondrial alterations in an animal model of obesity (*ob/ob* mice) and describe the beneficial effects of melatonin treatment on mitochondrial morphology and dynamics as influenced by mitofusin-2 and the intrinsic apoptotic cascade. Melatonin dissolved in 1% ethanol was added to the drinking water from postnatal week 5–13; the calculated dose of melatonin intake was 100 mg/kg body weight/day. Compared to control mice, obesity-related morphological alterations were apparent in the proximal tubules which contained round mitochondria with irregular, short cristae and cells with elevated apoptotic index. Melatonin supplementation in obese mice changed mitochondria shape and cristae organization of proximal tubules, enhanced mitofusin-2 expression, which in turn modulated the progression of the mitochondria-driven intrinsic apoptotic pathway. These changes possibly aid in reducing renal failure. The melatonin-mediated changes indicate its potential protective use against renal morphological damage and dysfunction associated with obesity and metabolic disease.

## Introduction

Obesity is considered an independent risk factor for chronic kidney disease, when associated with type 2 diabetes and hypertension [1], [2] and also in nondiabetic people [3], [4]. This condition is characterized by an excessive metabolic demand in adipose tissue and concomitant diabetic nephropathy [5]. Several lines of evidence highlight that excessive glomerular filtration with an increase in Bowman’s space is associated with an elevated glomerular hydrostatic pressure and that elevated proximal tubular sodium reabsorption plays a central role in the pathogenesis of glomerular hyperfiltration [6]. Although nephropathy in obese subjects is both common and complex and it is poorly understood. Moreover, few data are available on morphological changes that develop during proximal tubule damage [7]. Experimental data in animal models suggest that, in the diabetic kidney, the metabolic energy necessary to remove toxic deposits and to perform hyperfiltration by proximal tubules is deficient. This energy is provided by mitochondria and is essential for tubular-glomerular feedback [8].

Research has shown that mitochondrial damage and their functional disruption is due to increased generation of reactive oxygen species (ROS) and to mutations in proteins involved in the fusion-fission machinery. These represent an important pathogenic mechanism in renal diseases. Indeed, the morphology of mitochondria is related to the balance between fission and fusion [9]. Fission results in the formation of solitary organelles that may be easily removed by mitophagy or may be activated by the pro-apoptotic Bcl2 family machinery resulting in cytochrome c release and caspase activation and apoptosis. In contrast, the mitochondrial fusion machinery is associated with three GTPase dynamin-like proteins, named optic atrophy 1 (Opa1) and mitofusins (Mfn) 1 and 2 [10], [11]. In particular, Mfn2 is a transmembrane protein linked to the structural bridge between mitochondria and endoplasmic reticulum [12] and even through it may not be necessary for the juxtaposition of the organelles [13] it is related to calcium flux in metabolically active tissues. Mfn2, is closely associated to the development of diabetes, but its specific roles in the diabetic kidney remain unknown [5], [14].

Melatonin (N-acetyl-5-methoxytryptamine), a widely produced indoleamine and antioxidant [15]–[17], has been successfully used to limit metabolic alterations and mitochondrial dysfunctions in obese and in aged animals [18]–[22]. Emerging evidence has outlined the crucial role of melatonin in the prevention of oxidative mitochondrial damage due to its direct influence on free radical removal, cardiolipin peroxidation and cytochrome c release from mitochondria [23]–[25]. Moreover in metabolic diseases, such as obesity, inflammatory cytokines stimulate the generation of mitochondrial superoxide anion radicals and induce oxidative/nitrosative stress that causes defective aggregation of respiratory chain complexes. Melatonin neutralizes these changes and reduces radical mediated mitochondrial dysfunction [20], [26], [27].

Evidence has also shown an intimate interplay between misshaped mitochondria and altered mitochondrial bioenergetics. A mitochondrial reduction in membrane potential and fragmentation may be related to Mfn2 expression during obesity. These observations along with a reduction in the amplitude of the nocturnal pineal melatonin peak suggest that lower melatonin levels may be related to the mitochondrial deficiencies [10], [28], [29]. Thus, in the current study we carefully analyzed the mitochondrial structure in the proximal convoluted tubules of obese mice.

The aims of this study were to clarify mitochondrial alterations by investigating the roles of Mfn2 protein associated with convoluted tubule modifications in obese mice and to determine if melatonin would ameliorate or attenuate mitochondria dysfunction and the mitochondrial deformities. We also evaluated whether melatonin affects mitochondria-Mfn2-driven intrinsic apoptosis by an immunohistochemical analysis of specific markers such as cytochrome c, Bax and the caspase cascade (caspase 3 and caspase 9).

## Materials and Methods

### Animals and experimental protocols

Forty male mice, 20 lean and 20 obese (B6.V-Lep^ob^/OlaHsd), 4 weeks old were purchased from Harlan Laboratories S.r.l. (Udine, Italy) and divided into four groups (n = 10 mice/group) as follows: a) lean mice without treatment (lean); b) lean mice treated with melatonin for 8 weeks (lean+mel); c) obese mice without treatment (*ob/ob*); d) obese mice treated with melatonin for 8 weeks (*ob/ob*+mel). Melatonin (kindly provided by Chronolife S.r.l., Roma, Italy) was dissolved in 1% ethanol and diluted in the drinking water to yield a calculated final dose of 100 mg/kg body weight/day from postnatal week 5 to 13. The feeder jars were refilled daily with normal rodent chow, obtained from Harlan Laboratories S.r.l. (Udine, Italy) and the mean daily food intake were calculated by two observers blinded to the treatment. This study was carried out in strict accordance with the protocols of the Italian Ministry of Health and complied with “Guiding Principles in the Use of Animals in Toxicology” which were adopted by the Society of Toxicology in 1989. Protocols were approved by the Animal Care and Use Committee of the University of Brescia, Italy. Additional details about experimental procedures have been reported previously [18]. At the conclusion of postnatal week 13, all animals were killed by cervical dislocation and blood samples and kidneys were collected and processed for the serum analyses, the histological and immunofluorescence assays and for ultrastructural analyses.

### Analysis of serum and urine

Creatinine concentration in serum was determined using an enzymatic assay (Biovision, Milpitas, California, US), according to the manufacturers’ instructions.

Urine was collected in metabolic cages before death and 8-epiprostaglandin-F2α (8-epi-PGF2α) was calculated using an EIA kit (8-isoprostane; Cayman Chemical, Ann Arbor, MI, USA), according to the manufacturers’ instructions.

### Histopathology

One kidney was processed for histopathological staining and immunofluorescence assays. The tissue was fixed in 4% buffered paraformaldehyde for 24 hours, dehydrated in progressive ethanol solutions, xylene and embedded in paraffin wax, following the standard procedures. Subsequently, 7 µm-thick paraffin sections were cut stained using a Periodic acid-Schiff PAS solution to assess glomerular and tubular changes and mesangial proliferation [30]. On PAS stained sections at 400x magnification, two observers performed, in a blinded fashion, morphometric computerized analysis to evaluate glomerular area, using a light microscope (Olympus, Germany) equipped with an image analyzer (Image Pro Plus, Italy). In particular, 80 glomeruli, where vascular pole was evident, were examined in kidneys of each experimental group, as previously described [31].

### Transmission electron microscopy

The second kidney of each mouse was treated for ultrastructural analysis according to Rezzani et al. [32]. Briefly renal tissue was fixed by immersion in 2.5% glutaraldehyde in cacodylate buffer 0.1 M (pH 7.4) for 3 hours at +4°C and postfixed in 2% osmium tetroxide in cacodylate buffer for 1 hour at +4°C. Dehydration process was performed in increasing ethanol concentrations and propylene oxide followed by Araldite-Epon resin embedding. Semithin sections (1 µm-thick) were collected at an UltraCut E ultramicrotome stained by toluidine blue and observed at a light microscope (Olympus, Germany) to assess the presence of glomeruli and cortical tubules. Subsequently, from representative blocks, 70–80 nm-thick ultrathin sections were obtained using a diamond knife, collected on formvar coated grids, double stained with uranyl acetate and lead citrate and observed under a transmission electron microscope (Tecnai G2 Spirit) at 80 kV. Morphometric computerized analysis of the area of 100 mitochondria for each experimental group was blindly analyzed at 26.000x magnification by two observers blinded of the treatment.

### Immunofluorescence and immunohistochemistry assay

Alternate kidney sections were deparaffinized, rehydrated and incubated in 3% hydrogen peroxide, blocked with 1% bovine serum albumin (BSA) for 1 hour at room temperature, and then incubated overnight at 4°C with the following primary antibodies: mouse monoclonal antibody against mitofusin 2 (diluted 1∶600; H00009927-M01 Abnova, Taipei City, Taiwan); mouse monoclonal antibody against cytochrome c (diluted 1∶200; sc-13156 Santa Cruz Biotechnology Inc., Santa Cruz, CA, USA); rabbit polyclonal antibody against Bax (diluted 1∶400; sc-526 Santa Cruz Biotechnology Inc., Santa Cruz, CA, USA); mouse monoclonal antibody against caspase 3 (diluted 1∶400; sc-70497 Santa Cruz Biotechnology Inc., Santa Cruz, CA, USA) and rabbit polyclonal antibody against caspase 9 (diluted 1∶400; sc- 7885 Abcam, Cambridge, United Kingdom). After rinsing with phosphate buffered saline (PBS) the sections were labeled using goat anti-rabbit Alexa Fluor 546 (A11035) and goat anti-mouse or goat anti-rabbit Alexa Fluor 488 (A11029 and A11034 respectively) conjugated secondary antibodies (1∶200; Invitrogen, Paisley, United Kingdom). Finally, the sections were counter-stained with 4′,6-diamidino-2-phenylindole (DAPI), mounted and observed with a confocal microscope (LSM 510 Zeiss, Munich, Germany) [33].

For the immuohistochemical analysis, the sections were incubated in 3% hydrogen peroxide for 30 minutes, to inactivate the endogenous peroxidase activity. Then, after incubation in 1% BSA for 1 hour at room temperature, were incubated in mouse monoclonal antibody against mitofusin 2 (diluted 1∶200; H00009927-M01 Abnova, Taipei City, Taiwan) for 2 hours at 37°C. The sections were then sequentially incubated in anti-mouse biotinylated immunoglobulin and in avidin-biotin peroxidase complex. The reaction products were visualized using 0.33% hydrogen peroxide and 0.05% 3,3′-diaminobenzidine tetrahydrochloride as chromogen. The sections were finally counter-stained with haematoxylin, mounted and observed with a light microscope (Olympus, Germany).

The immunofluorescence and immunohistochemical controls were performed by omitting the primary antibody and in the presence of isotype matched IgGs.

Staining intensity of all the immunofluorescence assays was evaluated by two observers blinded to the treatments. The observers, using transmitted light, identified convoluted proximal tubules and then quantified the immunoposotivity. The evaluations were assumed to be correct if the values were not significantly different. In case of dispute concerning interpretation the case was reconsidered to reach a unanimous agreement.

In particular, the immunopositivity for mitofusin 2, cytochrome c, Bax, caspase 3 and caspase 9 were calculated, using an image analyzer (Image Pro Plus, Milan, Italy), for standardized areas, measuring 20 random fields with the same area for each experimental animals.

### Statistical analyses

The data were pooled to represent a mean value±standard deviation and statistical significance of differences among the experimental groups was evaluated by analysis of variance corrected by Bonferroni test with significance set at *p*<0.05. The levels of immunopositivity are expressed as arbitrary unit (AU).

## Results

All animals of each experimental group survived and melatonin supplementation in drinking water for 8 weeks was well tolerated. The food intake of the *ob/ob* animals was not significantly different from that of the lean group. Body and kidney weight for each animal was measured; weight parameters in *ob/ob* group were significantly higher compared those in control mice. We observed that melatonin supplementation to *ob/ob* mice did not influence significantly body or kidney weights, although the kidney/body weight ratio indicated proportional changes in the different experimental groups (Table 1).

**Table 1:** Food intake, body and kidney weights.

	*lean*	*lean plus melatonin*	*ob/ob*	*ob/ob plus melatonin*
**Food intake (g/day)**	4.45.±0.2	4.37±0.12	4.32±0.11	4.39±0.15
**Body weight (g)**	31.2±0.23	30.5±0.12	54.3±0.37	49.7±0.26
**Kidney weight (g)**	0.322±0.03	0.335±0.04	0.428±0.02	0.397±0.03
**Kidney/Body weight** **ratio (mg/g)**	10.27±0.18	10.86±0.22	8.21±0.16	7.65±0.18

Already at 13 weeks of age the obese mice showed an elevated value of serum creatinine respect to lean control mice, with or without melatonin treatment; this alteration of creatinine levels reflect loss of renal function. Moreover, renal oxidative stress, determined by urinary 8-isoprostane assay, was increased in obese mice compared to lean mice, with or without melatonin treatment Interestingly, melatonin treatment induced a significative reduction in serum creatinine and in urinary 8-isoprostane in the obese mice (Table 2).

**Table 2:** Creatinine and renal oxidative damage.

	*lean*	*lean plus melatonin*	*ob/ob*	*ob/ob plus melatonin*
**Creatinine (mg/dL)**	0.17±0.2	0.16±0.1	0.5±0.1	0.23±0.1
**8-isoprostane (pg/µmol)**	465±7	489±14	1153±25	576±18

**Figure 1:**
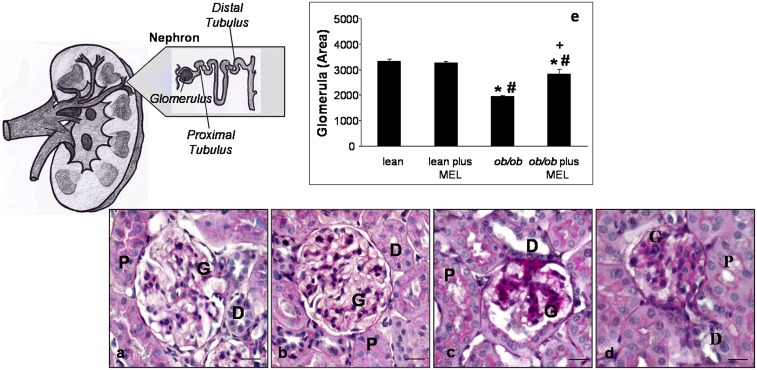
Histopathology. Photomicrographs showing PAS staining in the kidney of lean (a), lean mice treated with melatonin (b), *ob/ob* (c) and *ob/ob* treated with melatonin (d) mice. The graph summarizes the quantitative analyses of glomerular area for each experimental group (e). *p<0.05 *vs* lean, #*p*<0.05 *vs* lean plus melatonin and + *vs ob/ob*. (G) glomerulus; (P) proximal tubule and (D) distal tubule. Bar equals 20 µm.

**Figure 2:**
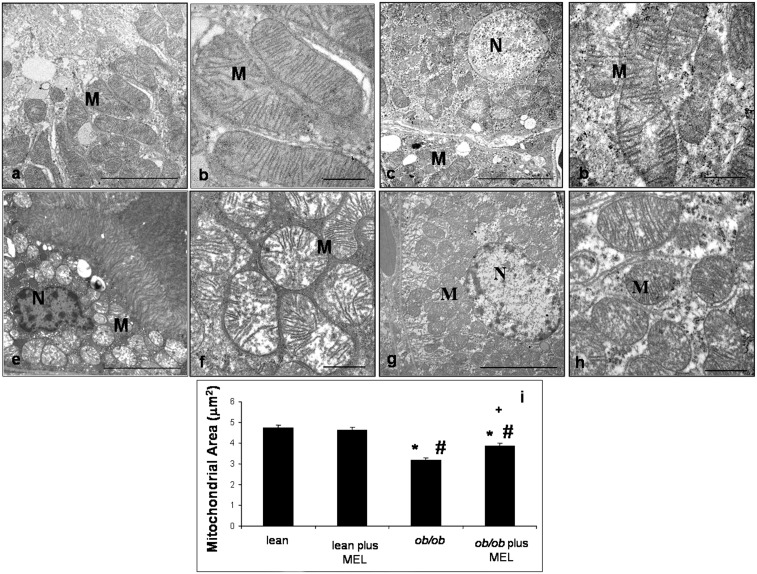
Transmission electron microscopy. Ultrastructural photomicrographs showing cortical proximal tubules and mitochondria of lean (a, b), lean mice treated with melatonin (c, d), *ob/ob* (e, f) and *ob/ob* treated with melatonin mice (g, h). The graph summarizes the quantitative analyses of mitochondrial area of each experimental groups (i). (M) mitochondria and (N) nucleus. Bar equals 1 µm. *p<0.05 *vs* lean, #*p*<0.05 *vs* lean plus melatonin and + *vs ob/ob*.

We next assessed renal morphology and proximal tubules ultrastructure. Normal features of glomeruli and cortical proximal renal tubules in lean mice, with or without melatonin treatment, were observed; in particular, proximal and distal tubular epithelium was regular with a PAS-positive brush border and central nuclei and showed a large lumen (Figure 1a, b). However, *ob/ob* mice showed irregular glomerular tufts with abundant mesangial matrix and a wide subcapsular space, an enlarged tubular epithelium, often devoid of a brush border, and a lumen filled with proteic aggregates in both proximal and distal tubules (Figure 1c). We observed that the renal histological architecture was partially restored after melatonin supplementation and that melatonin prevented major changes in proximal tubules where abnormal PAS-positive deposits disappeared and the brush border epithelium was preserved (Figure 1d). Moreover, quantitative data on total glomerular area, including both the glomerular tuft and subpodocytic space, documented that in *ob/ob* mice the total area decreased compared to that in lean mice, with or without melatonin treatment; in *ob/ob* mice that received melatonin, the total area was increased (about 15%) with respect to *ob/ob* non-treated group (*p*<0.05) (Figure 1e). The ultrastructural evaluation performed on cortical proximal tubules showed; in the lean group treated and untreated with melatonin, normal proximal tubular epithelial cells with regular apical microvilli and many mitochondria associated with basal membrane invaginations (Figure 2a, c). The mitochondria, in particular, appeared elongated with well-preserved regular cristae and a homogeneous inner matrix (Figure 2b, d). In contrast, proximal tubules of *ob/ob* mice presented apoptotic or necrotic nuclei and cytoplasm occupied by scattered round mitochondria (Figure 2e), that appeared hydropic, with irregular and peripherally-located short cristae (Figure 2f). After melatonin supplementation to the obese animals, mitochondria appeared elongated with more regular cristae (Figure 2g, h). Mitochondrial area was lower in *ob/ob* group with respect to that in lean mice and this was significantly increased after melatonin supplementation (Figure 2i).

**Figure 3:**
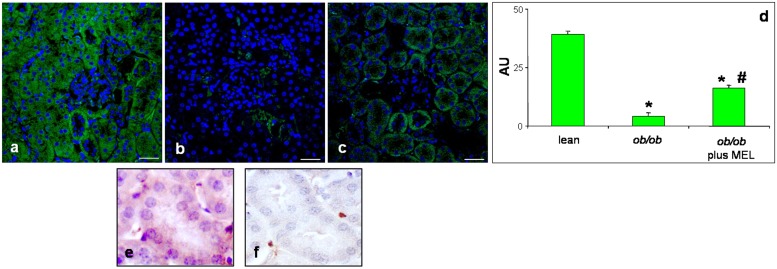
Mitofusin 2 immunofluorescence assay. Photomicrographs showing kidney mitofusin 2 immunostaining of lean (a, e), *ob/ob* (b, f) and ob/ob treated with melatonin (c) mice and relative quantitative analyses of immunopositivity (d). Bar equals 20 µm. *p<0.05 *vs* lean. Nuclei were stained with 4,6 diamidino-2-phenilindole. Photomicrographs of mitofusin 2 staining showing a cortical proximal tubule immunopositive (e) and an other one immunonegative (f).

The serum and urine assays and the morphological analyses did not reveal significantly differences between melatonin treated and non-melatonin treated control mice; thus, in immunofluorescence observations, they are considered without distinction (defined as lean mice). Immunofluorescent Mfn2 assay (green staining) showed that this mitochondrial marker was present as an intense basolateral signal in cortical tubules of lean mice (Figure 3a, e), whereas it was almost absent in renal proximal tubules of *ob/ob* mice (Figure 3b, f). After melatonin treatment, Mfn2 was partially preserved with a weak/moderate signal (Figure 3c). No expression or a very weak signal of Mfn2 was detected at glomerular level of each experimental group.

**Figure 4:**
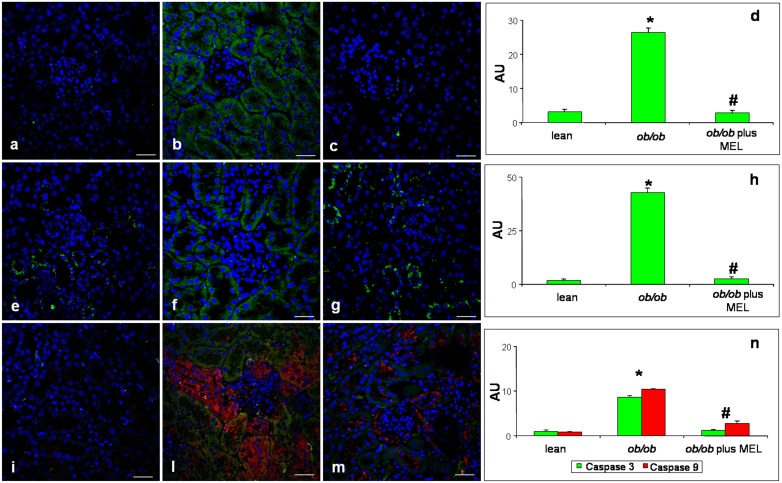
Apoptotic markers immunofluorescence assay. Photomicrographs of immunofluorescence analyses of kidney cytochrome c (green staining) of lean (a), *ob/ob* (b) and *ob/ob* treated with melatonin (c) mice. Immunofluorescence analyses of Bax (green staining) in kidney of lean (e), *ob/ob* (f) and *ob/ob* treated with melatonin (g) mice. Double immunofluorescence of caspase 3 (green staining) and caspase 9 (red staining) in kidney of lean (i), *ob/ob* (l) and *ob/ob* treated with melatonin (m) mice. Nuclei were stained with 4,6 diamidino-2-phenilindole. Bar equals 20 µm. The graphs summarize the quantitative analysis, respectively, of cytochrome c (d), Bax (h) and both caspase 3 and caspase 9 (n). *p<0.05 *vs* lean and #*p*<0.05 *vs ob/ob.*

Cytochrome c immunofluorescence staining (identified in green) was almost undetectable in lean mice (Figure 4a) and became evident, with a moderate/weak signal, in *ob/ob* mice (Figure 4b). No expression was detected at glomerular level of mice of either group. Moreover, also Bax immunofluorescence (green staining) showed absence or very weak expression in lean mice (Figure 4e) and a moderate/weak expression in cortical proximal tubules of *ob/ob* mice (Figure 4f). No expression was detected at the glomerula. Regarding caspases, their localization and expression (green staining for caspase 3 and red signal for caspase 9) were undetectable/very weak in lean mice (Figure 4i), whereas, in *ob/ob* mice both caspases became clearly expressed (moderate signal) in cortical tubules, but sometimes scattered in different sites. In particular, caspase 3 was prevalently localized in the proximal tubules, while caspase 9 was observed in the distal tubules (Figure 4l).

Moreover, we observed, after melatonin supplementation, that cytochrome c and also Bax were greatly attenuated (very weak/absent signal) due to melatonin treatment (Figure 4c, g) and also expression of both caspases decreased significantly in the proximal tubules (Figure 4m). Relative immunopositivity quantifications of each experimental group are plotted in figure 4d, h and n.

## Discussion

In the present study we showed that mitochondria of proximal tubules are morphologically altered in obese mice. Mnf2 expression was undetectable; while, staining of cytochrome c, Bax and capsases was evident in obese animals. Melatonin in the drinking water modified mitochondrial morphology and distribution in proximal tubules and greatly reduced specific markers of the intrinsic apoptotic pathway in kidneys of obese mice.

The obese mice developed an elevated serum creatinine already in the earliest phases of the pathogenesis, demonstrating that the onset of nephropathy is rapid and progressive. With regard to mitochondrial morphology, our findings are in agreement with several studies performed using muscles, liver and heart of obese animals [34], [35]. In our study, mitochondria of obese mice were roundish when compared to the elongated shape of mitochondria in control conditions. During ultrastructural analysis, cortical proximal tubules were found to have, in lean mice, elongated mitochondria, surrounded by a double membrane and with regular lamellar cristae. In contrast, in *ob/ob* mice, proximal tubular mitochondria were round with a few short cristae and with osmiophilic granules in the inner matrix. These findings, supported also by the observed increased in kidney 8-isoprostane, suggest that the mitochondria of the proximal convoluted tubules are significantly damaged in *ob/ob* mice, as a consequence of renal oxidative stress [36], [37].

To best define the mitochondrial morphological alteration in obesity, we observed a correlation between Mfn2 expression and the cytoarchitecture of mitochondria and their cristae in proximal tubules [5], [38]–[41]. Indeed, mitochondrial organization is commonly affected by a reduction in Mf2 in many organs including liver, heart and skeletal muscle, where this pleiotropic protein is associated with resistance to oxidative and mitochondrial DNA damage [41]–[43].

Recent studies indicate that, in healthy cells, Mfn2 is associated with the Bcl2 family proteins including Bax and Bak. This association in the cytosol allows maintenance of mitochondrial morphology during mitochondriogenesis [44]. During apoptosis, however, Bax translocates to the outer mitochondrial membrane (OMM), changes its conformation by becoming oligomeric, and alters its interaction with Mfn2 [45], [46]. Thus, here we analyzed the renal localization of Bax, and downstream pro-apoptotic markers including cytochrome c and caspases in *ob/ob* mice. While in control animals these signals were lacking, in the obese mice intense Bax and cytochrome c stainings were observed. It is known that Bax, upon oxidative damage, translocates to the OMM where it forms pore channels that allowed the flux of pro-apoptotic proteins from the inner mitochondrial membrane (IMM) to the cytosol, e.g. cytochrome c, Smac/Diablo, and the apoptosis inducing factor [47]. Moreover, in addition to opening the OMM pore, Bax remodels the cristae by interacting with specific proteins resident in the IMM [48], [49], in advance of the apoptosis cascade [50].

Regarding mitochondria-Mfn2-driven intrinsic apoptotic pathway, we detected the prevalence of caspase 3 in the proximal tubules and the caspase 9 signal in the distal tubules of *ob/ob* mice. To explain these observations, we noted that, among caspases, there are different roles with early initiator members, e.g. caspase 9, and later effector signals, such as caspase 3. When Bax in mitochondria triggers cytochrome c release, the apoptosome complex activates caspase 9 which further stimulates other downstream caspases, such as caspase 3. Moreover, the different distribution of the caspases as we observed may be due to the greater susceptibility of the proximal tubules to oxidative injury [51], [52] and resistance of the distal tubules to these processes which may adopt also an anaerobic metabolism [53].

Interestingly, melatonin restored creatinine levels, kidney oxidative stress and all damage linked to proximal tubules and especially mitochondria structure and Mfn2 expression. Melatonin is a potent antioxidant [17], [26], [54] and its actions on the mitochondrial alterations strongly indicates that the modification on these organelles were related to ROS production [55] as also occurs during aging [56]. These results are supported also by other studies showing similar morphological changes in muscle mitochondria of streptozotocin-treated mice and effects of N-acetylcysteine, another antioxidant [36]. In obese mice melatonin clearly attenuated mitochondria-driven apoptosis; this observation is in line with that of Radogna et al. [57], who showed a direct antagonistic effect of melatonin on Bax activation at the mitochondrial level during an apoptotic challenge. Moreover, we showed that melatonin strongly limited the caspase cascade expression in obese kidneys consistent with its anti-apoptotic activities observed by Molpeceres et al. (2007) [58].

**Figure 5:**
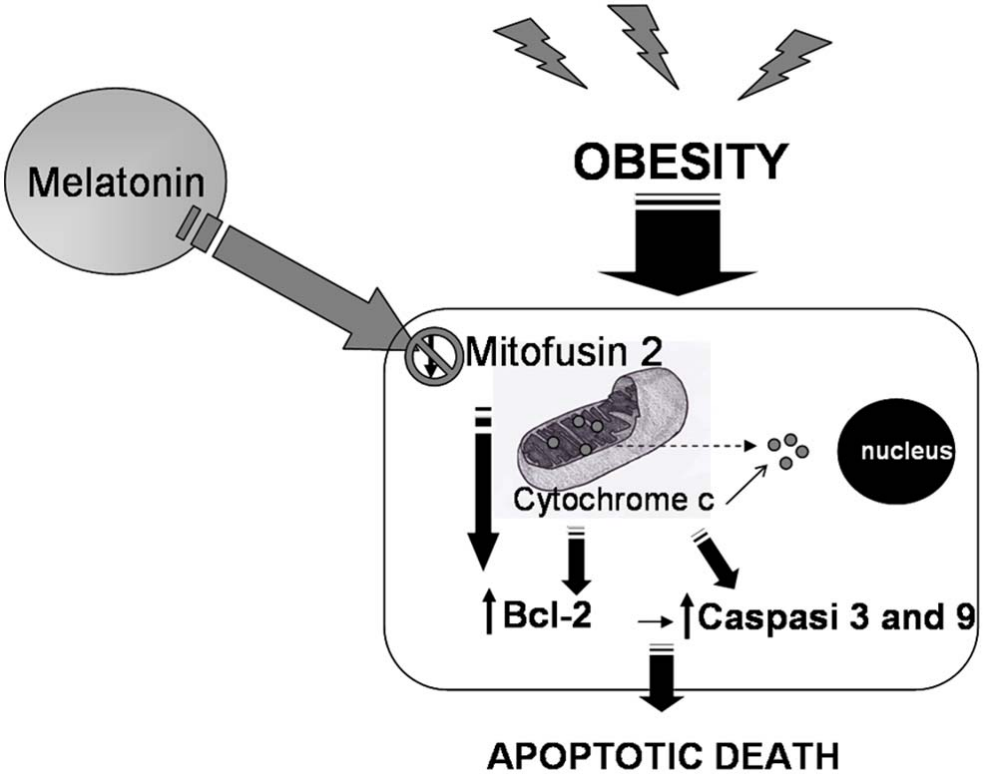
Melatonin remodels mitochondria in obese mice kidneys. A schematic representation of the apoptotic pathway induced by obesity mediated alterations in the kidney conditioned by upstream mitofusin 2 and the inhibiting action of melatonin on the mitochondria-driven intrinsic apoptotic cascade.

Our study would stimulate the attention of scientists on the role of melatonin in the kidney of obese mice, specifically in terms of its ability to remodel mitochondrial structure and to restore Mfn2 in cortical proximal tubules. Via these crucial events, melatonin may negatively influence mitochondrial Bax sensitization and consequently cytochrome c efflux, thereby abrogating the caspase-dependent intrinsic mitochondrial apoptotic program (Figure 5).
